# Supplementary material for: A framework to identify opportunities to address socioscientific issues in the elementary school curricula: A case study from England, Italy, and Portugal
Source: PLoS One. 2025 Mar 19;20(3):e0308901. doi: 10.1371/journal.pone.0308901 (PMC11957555; doi:10.1371/journal.pone.0308901)
Supplement: S1 Table — (DOCX) [file pone.0308901.s001.docx]

S1 Table - Initial framework of analysis

| **Category** | **Subcategories** | **Sub-subcategories** | **Guidelines** |
| --- | --- | --- | --- |
| Awareness of the issue | Biotechnology | Medicine | Include in this subcategory all biotechnology-related problems that have their applications in the medical field |
|  |  | Agronomy | Include in this sub-category all biotechnology-related problems that have their application in the field of agriculture |
|  |  | Environmental | Include in this subcategory all biotechnology-related problems that have their application in the environmental field |
|  |  | Genetics | Include in this subcategory all biotechnology-related problems that have their application in the field of genetics |
|  |  | Industry | Include in this subcategory all biotechnology-related problems that have their applications in the field of industry |
|  | Health issues | Diseases | Include in this subcategory all problems that have their origin related to a disease or the treatment of a disease |
|  |  | Food | Include in this subcategory all health-related problems that have their origin in food production or consumption |
|  |  | Resources | Include in this subcategory all health-related problems that have as their origin the scarcity of resources or problems of access to those resources |
|  |  | Use of medicines | Include in this subcategory all health-related problems that have their origin in the incorrect use of medicines |
|  | Environmental issues | Energy production | Include in this subcategory all environmental problems arising from energy production, distribution and use |
|  |  | Pollution | Include in this subcategory all environmental problems arising from pollution |
|  |  | - Waste-related | Include in this subcategory all problems arising from pollution that are related to waste production |
|  |  | - Atmospheric | Include in this subcategory all pollution-related problems that have the greatest impact on air pollution |
|  |  | Consumption | Include in this subcategory all environment-related problems that have their origin in the production and consumption of goods |
|  |  | Biodiversity | Include in this subcategory all environment-related issues that have impacts on biodiversity conservation |
|  | Exobiology issues | - | Include in this category all problems that have exobiology as their main focus or origin |
| **Category** | **Subcategory** | **Guidelines** | |
| Socioscientific reasoning | Account for the inherent complexity of SSI | Include in this subcategory all learning objectives connected with socioscientific reasoning, specifically, those related to the inherent complexity of SSI. | |
|  | Analyze issues from multiple perspectives | Include in this subcategory all learning objectives connected with socioscientific reasoning, specifically, those related to analyzing issues from multiple perspectives. | |
|  | Identify aspects of issues that are subject to ongoing inquiry | Include in this subcategory all learning objectives connected with socioscientific reasoning, specifically, those related to identifying issues that are subject to ongoing inquiry. | |
|  | Employ skepticism in analysis of potentially biased information | Include in this subcategory all learning objectives connected with socioscientific reasoning, specifically, those related to employing skepticism in analysis of potentially biased information. | |
|  | Explore how science can contribute to the issues and the limitations of science | Include in this subcategory all learning objectives connected with socioscientific reasoning, specifically, those related to exploring how science can contribute to the issues and the limitations of science. | |
| **Category** | **Guidelines** | | |
| Identity | Include in this subcategory all learning objectives related to Identity.  By working on identity, students: i) position themselves with new competencies, interests, and ideas about themselves that enable new patterns of participation and discourse; ii) engage with complex SSI both in and out of school; iii) develop an interest in contributing to discourses about complex issues in society, but also see themselves as valuable contributors to those discourses.  Identity development is a learning goal that transcends single teaching and learning experiences or units. | | |
