# Supplementary material for: A framework to identify opportunities to address socioscientific issues in the elementary school curricula: A case study from England, Italy, and Portugal
Source: PLoS One. 2025 Mar 19;20(3):e0308901. doi: 10.1371/journal.pone.0308901 (PMC11957555; doi:10.1371/journal.pone.0308901)
Supplement: S2 Table — EN - English school curriculum, IT - Italian school curriculum, PT - Portuguese school curriculum. IT and PT learning objectives were translated by the authors. (i) Subcategories and sub-subcategories that emerged from the inductive analysis. (DOCX) [file pone.0308901.s002.docx]

S2 Table - Framework for Identifying Opportunities to implement the SSI approach in science school curricula (FIOSSI)

| **Category** | **Subcategories** | **Sub-subcategories** | **Guidelines** | **Examples*** |
| --- | --- | --- | --- | --- |
| 1.Awareness of the issue | 1.1.Technology issues^(i)^ | 1.1.0 Technology issues in general^(i)^ | Include here issues that are related to the technology category but for which no association with specific sub-subcategories is possible. | EN, 6th grade - Compare and give reasons for variations in how components function, including the brightness of bulbs, the loudness of buzzers and the on/off position of switches  PT, 1st grade - Recognize that technology responds to everyday needs and problems (electricity grid, water pipes, telecommunications, etc.) |
|  |  | 1.1.1 Medicine | Include in this sub-subcategory all technology-related issues that have their application in the medical field. | PT, 4th grade - Recognize the importance of technological evolution for the evolution of society, associating objects, equipment and technological solutions with different needs and problems of everyday life (prediction/mitigation of natural and technological disasters, health, telecommunications, transportation, etc.) |
|  |  | 1.1.2 Agronomy/Agriculture | Include in this sub-subcategory all technology-related issues that have their application in the agronomy/agriculture field. |  |
|  |  | 1.1.3 Environment | Include in this sub-subcategory all technology-related issues that have their application in the environmental field. | PT, 4th grade - Recognize the importance of technological evolution for the evolution of society, associating objects, equipment and technological solutions with different needs and problems of everyday life (prediction/mitigation of natural and technological disasters, health, telecommunications, transportation, etc.) |
|  |  | 1.1.4 Genetics | Include in this sub-subcategory all technology-related issues that have their application in the genetics field. |  |
|  |  | 1.1.5 Industry | Include in this sub-subcategory all technology-related issues that have their application in the industry field. |  |
|  |  | 1.1.6 Animal experiments^(i)^ | Include in this sub-subcategory all technology-related issues where the use of animals is the main issue. |  |
|  |  | 1.1.7 Information and Communications Technology (ICT)^(i)^ | Include in this sub-subcategory all technology-related issues in which the use of Internet, Information and Communications Technology is involved and controversial (e.g.: social media, profiling, privacy, ...). | PT, 4th grade - Use information and communication technologies safely, respectfully, and responsibly, becoming aware that their abusive use generates dependence (games, social networks, etc.) |
|  | 1.2. Human health issues | 1.2.0 Human health issues in general^(i)^ | Include here issues that are related to the human health issues category but which no association with specific sub-subcategories is possible. | EN, 3rd grade - Recognise that light from the sun can be dangerous and that there are ways to protect their eyes  IT, 3rd grade - Observe and pay attention to the functioning of one's own body (hunger, thirst, pain, movement, cold and heat, etc.) in order to recognise it as a complex organism, proposing elementary models of its functioning  PT, 4th grade - Describe, in a simplified way, and using representations, the digestive, respiratory, circulatory, excretory and reproductive systems, recognizing that their proper functioning requires specific care |
|  |  | 1.2.1 Diseases | Include in this sub-subcategory all issues that have their origin connected to a disease or the treatment of a disease. | EN, 2nd grade - Describe the importance for humans of exercise, eating the right amounts of different types of food, and hygiene  PT, 6th grade - Explore how microorganisms can cause harm to humans |
|  |  | 1.2.2 Food | Include in this sub-subcategory all health-related issues that result from food production or consumption. | EN, 3rd grade - Identify that animals, including humans, need the right types and amount of nutrition, and that they cannot make their own food; they get nutrition from what they eat  IT, 5th grade - Take care of one's own health also from a nutritional and physical point of view  PT, 6th grade - Elaborar algumas ementas equilibradas e discutir os riscos e os benefícios dos alimentos para a saúde humana |
|  |  | 1.2.3 Access to resources | Include in this sub-subcategory all health-related issues that have their origin in access to resources or their scarcity. | EN, 2nd grade - Find out about and describe the basic needs of animals, including humans, for survival (water, food and air)  PT, 5th grade - Interpret water bottle labels and justify the importance of water to human health |
|  |  | 1.2.4 Use of medicines | Include in this sub-subcategory all health-related issues that have their origin in the use of medicines. | EN, 6th grade - Recognise the impact of diet, exercise, drugs and lifestyle on the way their bodies function  PT, 2nd grade - Recognize the importance of vaccination and the correct use of medicines, particularly antibiotics |
|  |  | 1.2.5 Human diversity^(i)^ | Include in this sub-subcategory all health-related issues that arise from human diversity (e.g.: gender differences or equality, reproductive issues, skin color, food intolerances, ...)  Note: this sub-subcategory is different from the sub-subcategory 'Biological diversity' of subcategory 5, which is intended for issues that derive from biological diversity but generate social conflicts and biases. This sub-subcategory refers to human diversity related to health issues. | IT, 5th grade - Acquire initial information on reproduction and sexuality  PT, 4th grade - Know some biological and behavioral changes that occur during adolescence |
|  |  | 1.2.6 Non-nutrient substances intake^(i)^ | Include in this sub-subcategory all health-related issues that have their origin in the intake of non-nutrient substances (e.g.: smoke, drugs, alcoholic substances, …). | EN, 6th grade - Recognise the impact of diet, exercise, drugs and lifestyle on the way their bodies function  PT, 3rd grade - Link everyday habits with healthy lifestyles, recognizing that alcohol, tobacco, and other drug use is harmful to health |
|  | 1.3. Environmental issues | 1.3.0 Environmental issues in general^(i)^ | Include here issues that are related to the environmental issues category but which no association with specific sub-subcategories is possible. | EN, 4th grade - Recognise that environments can change and that this can sometimes pose dangers to living things  IT, 3rd grade - Recognise in other living organisms, in relation to their environments, needs similar to one's own  PT, 3rd grade - Recognize how environmental changes (deforestation, fires, silting, pollution) cause imbalances in ecosystems and influence the lives of living beings (survival, death, and migration) and society |
|  |  | 1.3.1 Energy | Include in this sub-subcategory all environmental issues arising from energy production, distribution and use. | EN, 4th grade - Recognise some common conductors and insulators, and associate metals with being good conductors  PT, 2nd grade - Know how to ask questions about environmental problems existing in the locality where they live, particularly those related to water, energy, waste, air, soils, presenting proposals for intervention |
|  |  | 1.3.2 Pollution | Include in this sub-subcategory all environmental issues arising from pollution (only if they cannot be included in sub-subcategories 3.2.1, 3.2.2, 3.2.3 & 3.2.4). | PT, 3rd grade - Identify an environmental or social problem existing in your community (urban solid waste, pollution, poverty, unemployment, social exclusion, etc.), proposing solutions |
|  |  | 1.3.2.1 Waste-related | Include in this sub-subcategory all issues arising from pollution that are related to waste production. | PT, 4th grade - Produce technological solutions by reusing or recycling materials (windmill, solar oven, etc) |
|  |  | 1.3.2.2 Atmospheric | Include in this sub-subcategory all pollution-related issues that have the greatest impact on air. | PT, 5th grade - Argue about the impacts of human activities on air quality and about measures that contribute to its preservation, with local, regional, national or global examples and integrating knowledge from other disciplines |
|  |  | 1.3.2.3 Water^(i)^ | Include in this sub-subcategory all pollution-related issues that have the greatest impact on water. | PT, 5th grade - Distinguish water fit for consumption (potable and mineral) from water unfit for consumption (brackish and polluted), analyzing local, regional or national problematic issues |
|  |  | 1.3.2.4 Soil^(i)^ | Include in this sub-subcategory all issues related to soil degradation and/or pollution. |  |
|  |  | 1.3.3 Consumerism | Include in this sub-subcategory all environmental issues arising from the production and consumption of goods. | PT, 5th grade - Discuss the importance of sustainable water management in terms of its use, exploitation and protection, with local, regional, national or global examples |
|  |  | 1.3.4 Biodiversity | Include in this sub-subcategory all environmental issues that have impacts on biodiversity conservation. | EN, 2nd grade - Identify that most living things live in habitats to which they are suited and describe how different habitats provide for the basic needs of different kinds of animals and plants, and how they depend on each other  PT, 5th grade - Identify invasive flora and fauna species and their consequences for local biodiversity |
|  |  | 1.3.5 Territory management^(i)^ | Include in this sub-subcategory all environmental issues arising from territory management. | PT, 4th grade - Recognize how human activity interferes with the ocean (pollution, changes in coastal areas and rivers, etc.) |
|  | 1.4. Exobiology issues | - | Include in this sub-subcategory all exobiology issues arising from the study of the origin, early evolution, distribution, and future of life in the universe. |  |
|  | 1.5. Social conflicts and biases based on human diversity issues^(i)^ | 1.5.0 Social conflicts and biases based on human diversity issues in general^(i)^ | Include here issues that are related to the social conflicts and biases based on human diversity category but for which no association with specific sub-subcategories is possible. | PT, 1st grade - Promote strategies that require/induce from the student respect for individual differences |
|  |  | 1.5.1 Biological diversity^(i)^ | Include in this sub-subcategory all issues that have their origin in human biological diversity (e.g.: skin color, gender differences, ...).  Note: this sub-subcategory is different from the sub-subcategory 'Human diversity' of subcategory 2, which is intended for health issues. This sub-subcategory refers to issues that derive from biological diversity but generate social conflicts and biases. | EN, overall curriculum - Teachers should take account of their duties under equal opportunities legislation that covers race, disability, sex, religion or belief, sexual orientation, pregnancy and maternity, and gender reassignment  PT, 3rd grade - Recognize that living things reproduce and that their offspring have similar characteristics to their parents, but also differ in some of them |
|  |  | 1.5.2 Socio-cultural diversity^(i)^ | Include in this sub-subcategory all issues that have their origin in human socio-cultural differences (e.g.: economical situation, historical development, religious beliefs, cultural habits, ...). | EN, overall curriculum - Teachers must also take account of the needs of pupils whose first language is not English. Monitoring of progress should take account of the pupil’s age, length of time in this country, previous educational experience and ability in other languages  PT, 5th grade - Promote strategies that require/induce from the student respect for differences in characteristics, beliefs, or opinions |
| **Category** | **Subcategory** | **Guidelines** | | **Examples** |
| 2.Socioscientific reasoning | 2.0 Socioscientific reasoning in general^(i)^ | Include in this subcategory all learning objectives related with socioscientific reasoning but for which no association with specific subcategories is possible.  Note: socioscientific reasoning practices are those that engage learners in practices that reflect the social and scientific intersections that make the focal issue complex, interesting, and difficult to resolve.  This category is more concerned with skills. | | EN, general aims - Are equipped with the scientific knowledge required to understand the uses and implications of science, today and for the future  PT, 6th grade - Promote strategies that require the student to know how to question a situation |
|  | 2.1 Account for the inherent complexity of SSI | Include in this subcategory all learning objectives connected with socioscientific reasoning, specifically, those related to the inherent complexity of SSI. | | IT, 5th grade - Pursue the observation and interpretation of environmental changes, including global changes, particularly those resulting from modifications due to humans  PT, 1st to 4th grade - Identify natural, social, and technological elements of the environment and their interrelationships |
|  | 2.2 Analyze issues from multiple perspectives | Include in this subcategory all learning objectives connected with socioscientific reasoning, specifically, those related to analyzing issues from multiple perspectives.  Note: consider multiple perspectives as individual, social or disciplinary perspectives. | | EN, general aims - They must be assisted in making their thinking clear, both to themselves and others, and teachers should ensure that pupils build secure foundations by using discussion to probe and remedy their misconceptions  IT, 5th grade - The student finds from various sources (books, internet, adult discourse, etc.) information and explanations on problems that interest her/him  PT, 1st grade - Promote strategies that require/induce by the student confrontation of ideas about the approach to a given problem and/or the way to solve it |
|  | 2.3 Identify aspects of issues that are subject to ongoing inquiry | Include in this subcategory all learning objectives connected with socioscientific reasoning, specifically, those related to identifying issues that are subject to ongoing inquiry. | | EN, 3rd and 4th grades - Using results to draw simple conclusions, make predictions for new values, suggest improvements and raise further questions |
|  | 2.4 Employ skepticism in analysis of potentially biased information | Include in this subcategory all learning objectives connected with socioscientific reasoning, specifically, those related to employing skepticism in analysis of potentially biased information.  Note: consider also learning objectives related to the development of critical thinking, as this includes the component skills of analyzing arguments, making inferences using inductive and/or deductive reasoning, judging or evaluating, and making decisions or solving problems. | | EN, 5th and 6th grades - Taking measurements, using a range of scientific equipment, with increasing accuracy and precision, taking repeat readings when appropriate  IT, intro - Experimental research, both individual and in groups, strengthens children's confidence in their own thinking skills, willingness to give and receive help, learning from their own and others' mistakes, openness to different opinions and the ability to argue their own  PT, 2nd grade - Promote strategies that develop students' critical and analytical thinking, focusing on exposing reasons that support assertions |
|  | 2.5 Explore how science can contribute to the issues and understand the limitations of science in issue resolution | Include in this subcategory all learning objectives connected with socioscientific reasoning, specifically, those related to exploring how science can contribute to the issues and to understanding the limitations of science in issue resolution.  Note: consider technology as a scientific field in this category. | | EN, general aims - They should be encouraged to understand how science can be used to explain what is occurring, predict how things will behave, and analyze causes  PT, 5th grade - It is also important that students understand that science is present in our daily lives and that more and more scientific and technological knowledge is needed to assume a citizenship perspective, live with quality of life and contribute to the sustainability of planet Earth |
| **Category** | **Guidelines** | | | **Examples** |
| 3.Socioscientific identity | (code number - 3)  Include in this category all learning objectives related to socioscientific identity. By working on identity students:  i) develop predisposition to approach SSI using Socioscientific reasoning skills^(i)^;  ii) engage with complex SSI both in and out of school;  iii) develop an interest in contributing to discourses about complex issues in society, but also see themselves as valuable contributors to those discourses.  Note: this category is more concerned with attitudes. | | | EN, overall curriculum - Promotes the spiritual, moral, cultural, mental and physical development of pupils at the school and of society  IT, 5th grade - The student develops attitudes of curiosity and ways of looking at the world that stimulate her/him to seek explanations for what she/he sees happening  PT, 5th grade - Assume attitudes and values that advocate the implementation of measures aimed at promoting the sustainability of planet Earth and fostering individual and collective health |

Legend: * The examples provided are learning objectives from the analyzed curricula. EN - English school curriculum, IT - Italian school curriculum, PT - Portuguese school curriculum. IT and PT learning objectives were translated by the authors. ^(i)^ Subcategories and sub-subcategories that emerged from the inductive analysis.
