# Supplementary material for: A framework to identify opportunities to address socioscientific issues in the elementary school curricula: A case study from England, Italy, and Portugal
Source: PLoS One. 2025 Mar 19;20(3):e0308901. doi: 10.1371/journal.pone.0308901 (PMC11957555; doi:10.1371/journal.pone.0308901)
Supplement: S3 Table — fr (%) - relative frequency within each country (English - N = 78, Italian - N = 18, Portuguese - N = 313, with N meaning the total number of evidence found in each country) (DOCX) [file pone.0308901.s003.docx]

S3 Table - Distribution of opportunities for SSI found in the three curricula analyzed according to the structure of each curriculum.

| English | | Italian | | Portuguese | |
| --- | --- | --- | --- | --- | --- |
| **Curricula structure** | **fr (%)** | **Curricula structure** | **fr (%)** | **Curricula structure** | **fr (%)** |
| Overall curriculum | 14.10% | Introduction | 16.67% | General aims for grades 1 to 4 | 4.79% |
| General aims | 17.95% |  |  |  |  |
| Key stage 1 (learning goals for grades 1 and 2) | 6.41% | Grades 1 to 3 | 22.22% | Grade 1 | 8.31% |
| Grade 1 | 1.28% |  |  |  |  |
| Grade 2 | 11.54% |  |  | Grade 2 | 11.82% |
| Lower key stage 2 (learning goals for grades 3 and 4) | 12.82% |  |  | Grade 3 | 15.34  % |
| Grade 3 | 3.85% |  |  |  |  |
| Grade 4 | 5.13% | Grades 4 to 5 | 61.11% | Grade 4 | 17.25% |
| Upper key stage 2 (learning goals for grades 5 and 6) | 16.67% |  |  | General aims for grades 5 to 6 | 1.28% |
| Grade 5 | 1.28% |  |  | Grade 5 | 19.49% |
| Grade 6 | 8.97% |  |  | Grade 6 | 21.73% |

Legend: fr (%) - relative frequency within each country (English - N=78, Italian - N=18, Portuguese - N=313, with N meaning the total number of evidence found in each country)
