# Supplementary material for: A framework to identify opportunities to address socioscientific issues in the elementary school curricula: A case study from England, Italy, and Portugal
Source: PLoS One. 2025 Mar 19;20(3):e0308901. doi: 10.1371/journal.pone.0308901 (PMC11957555; doi:10.1371/journal.pone.0308901)
Supplement: S4 Table — (DOCX) [file pone.0308901.s004.docx]

S4 Table - Absolute frequencies of the learning goals attributed to a FIOSSI category, subcategory and sub-subcategory per school curriculum (see the definition of FIOSSI categories, subcategories and sub-subcategories in S2 Table)

| Category | Subcategory and sub-subcategory | English school curriculum | Italian school curriculum | Portuguese school curriculum |
| --- | --- | --- | --- | --- |
| 1 |  | 1 | 0 | 18 |
| 1 | 10 | 1 | 0 | 10 |
| 1 | 11 | 0 | 0 | 1 |
| 1 | 12 | 0 | 0 | 0 |
| 1 | 13 | 0 | 0 | 1 |
| 1 | 14 | 0 | 0 | 0 |
| 1 | 15 | 0 | 0 | 0 |
| 1 | 16 | 0 | 0 | 0 |
| 1 | 17 | 0 | 0 | 6 |
| 2 |  | 17 | 5 | 47 |
| 2 | 20 | 7 | 3 | 23 |
| 2 | 21 | 1 | 0 | 7 |
| 2 | 22 | 5 | 1 | 6 |
| 2 | 23 | 2 | 0 | 2 |
| 2 | 24 | 1 | 0 | 2 |
| 2 | 25 | 0 | 1 | 6 |
| 2 | 26 | 1 | 0 | 1 |
| 3 |  | 8 | 5 | 60 |
| 3 | 30 | 5 | 5 | 14 |
| 3 | 31 | 1 | 0 | 1 |
| 3 | 32 | 0 | 0 | 6 |
| 3 | 321 | 0 | 0 | 2 |
| 3 | 322 | 0 | 0 | 1 |
| 3 | 323 | 0 | 0 | 1 |
| 3 | 324 | 0 | 0 | 0 |
| 3 | 33 | 0 | 0 | 11 |
|  | 34 | 2 | 0 | 16 |
| 3 | 35 | 0 | 0 | 8 |
| 4 | 40 | 0 | 0 | 0 |
| 5 |  | 12 | 0 | 29 |
| 5 | 50 | 0 | 0 | 11 |
| 5 | 51 | 5 | 0 | 4 |
| 5 | 52 | 7 | 0 | 14 |
| 6 |  | 36 | 4 | 124 |
| 6 | 60 | 7 | 0 | 24 |
| 6 | 61 | 0 | 1 | 1 |
| 6 | 62 | 12 | 2 | 56 |
| 6 | 63 | 5 | 0 | 0 |
| 6 | 64 | 6 | 1 | 31 |
| 6 | 65 | 6 | 0 | 12 |
| 7 | 70 | 4 | 4 | 35 |
| Total | | 78 | 18 | 313 |
